# Evolution of Surgical Treatment of Colorectal Liver Metastases in the Real World: Single Center Experience in 1212 Cases

**DOI:** 10.3390/cancers13051178

**Published:** 2021-03-09

**Authors:** Francesca Ratti, Federica Cipriani, Guido Fiorentini, Valentina Burgio, Monica Ronzoni, Angelo Della Corte, Stefano Cascinu, Francesco De Cobelli, Luca Aldrighetti

**Affiliations:** 1 Hepatobiliary Surgery Division, IRCCS San Raffaele Scientific Institute, 20132 Milan, Italy; cipriani.federica@hsr.it (F.C.); fiorentini.guido@hsr.it (G.F.); aldrighetti.luca@hsr.it (L.A.); 2 Department of Medical Oncology, IRCCS San Raffaele Scientific Institute, 20132 Milan, Italy; burgio.valentina@hsr.it (V.B.); ronzoni.monica@hsr.it (M.R.); cascinu.stefano@hsr.it (S.C.); 3 Experimental Imaging Center, Radiology Department, IRCCS San Raffaele Scientific Institute, 20132 Milan, Italy; dellacorte.angelo@hsr.it (A.D.C.); decobelli.francesco@hsr.it (F.D.C.)

**Keywords:** colorectal metastases, outcome, trends, laparoscopy, oncology

## Abstract

**Simple Summary:**

The main purpose of this study is to evaluate the evolution of surgical treatment of CRLM within a referral center, comparing three consecutive time periods. Trends will be assessed with a specific focus on technical issues, such as the adoption of the laparoscopic approach and strategies to induce liver hypertrophy, as well as oncological issues, such as the variation in characteristics of the disease (Clinical Risk Score and extrahepatic metastases) and long term results. The secondary endpoint will be to evaluate, through uni- and multivariate analysis, the predictive factors for inclusion to a minimally invasive approach (technical issue) and the predictive factors of overall survival (oncological issue). Results demonstrated that, within the study period, the cultural background, the maturation of technical expertise and the consolidation of the multidisciplinary team have resulted in safe expansion of the possibility to offer a curative opportunity to patients, while continuously implementing into clinical practice evidence provided by the literature.

**Abstract:**

Background: In recent years, the treatment of colorectal liver metastases (CRLM) has undergone significant evolution thanks to technical improvements as well as oncological advances, which have been the subject of targeted studies aimed at understanding the details of this heterogeneous disease. The purpose of this study is to put together pieces of this complex scenario by providing an overview of the evolution that has occurred in the context of a single center within a multidisciplinary management approach. Methods: Between 2005 and 2020, 1212 resections for CRLM were performed at the Hepatobiliary Surgery Division of San Raffaele Hospital, Milan. The series was divided into three historical periods, which were compared in terms of disease characteristics and short- and long-term outcomes: Period 1, 2005–2009 (293 cases); Period 2, 2010–2014 (353 cases); Period 3, 2015–2020 (566 cases). The trends for surgical technical complexity, oncological burden of the disease, use of the laparoscopic approach and use of techniques for hepatic hypertrophy were analyzed year by year. Uni- and multivariate analyses were performed to identify factors associated with inclusion to a laparoscopic approach and with long-term prognosis. Results: The number of resections performed over the years progressively increased, with an increase in the number of cases with a high Clinical Risk Score and a high profile of technical complexity. The proportion of cases performed laparoscopically increased, but less rapidly compared to other malignant tumors. The risk of postoperative morbidity and mortality was similar in the three analyzed periods. Long-term survival, stratified by Clinical Risk Score, improved in Period 3, while overall survival remained unchanged. Conclusion: The cultural background, the maturation of technical expertise and the consolidation of the multidisciplinary team have resulted in safe expansion of the possibility to offer a curative opportunity to patients, while continuously implementing into clinical practice evidence provided by the literature.

## 1. Introduction

The potentially curative role of surgery for ColoRectal cancer Liver Metastases (CRLM) has become increasingly recognized and widely reported in the modern era [1,2]. In particular, the favourable synergistic combination of surgery and chemotherapy [3,4] has led to a significant evolution of clinical practices in recent years, attributed primarily to the overall increased expected survival on one hand [5] and to the expansion of criteria for enrolment to surgery on the other [6].

In parallel with this evolving scenario, the scientific literature has concomitantly focused on specific technical [7,8,9,10,11,12,13] and therapeutic aspects [13,14,15,16,17,18,19] of management of CRLM.

This multitude and diversity of issues is representative of the inherently heterogeneous nature of CRLMs, where drastically divergent scenarios are the rule rather than the exception within this single disease entity. In this regard, multidisciplinary management has always remained central to outlining and coordinating the optimal therapeutic strategy [20,21].

The specificity of trials and the frequent need to resort to multicentre studies to adjust for sample size and provide stronger levels of evidence, however, does not provide a clear overview of the evolution of CRLM surgery over recent years within a tertiary referral center, where human resources have been remained stable while the expertise and cultural background of personnel have evolved.

The main purpose of this study is to evaluate the evolution of surgical treatment of CRLM within a referral center, comparing three consecutive time periods. Trends will be assessed with a specific focus on technical issues, such as the adoption of the laparoscopic approach and strategies to induce liver hypertrophy, as well as oncological issues, such as the variation in characteristics of the disease (Clinical Risk Score and extrahepatic metastases) and long-term results.

The secondary endpoint will be to evaluate, through uni- and multivariate analysis, the predictive factors for inclusion to a minimally invasive approach (technical issue) and the predictive factors of overall survival (oncological issue).

## 2. Methods

### 2.1. Study Design

Between January 2005 and March 2020, 3322 liver resections were performed at the Hepatobiliary Surgery Division of San Raffaele Hospital, Milan. Data from these procedures was collected in a prospective database and reviewed retrospectively. During the study period, 1212 resections for CRLM (36.5% of the institutional series of liver resections) were performed and these constituted the population of the present study. Procedures with any of the following characteristics were excluded from analysis: unconfirmed diagnosis (at final pathology) of liver metastases from colonic or rectal adenocarcinoma; surgical ablations of CRLMs without concurrent liver resection; less than 6 months of follow up. The series was divided into three historical periods, which were compared in terms of disease characteristics and short- and long-term outcomes: Period 1, 2005–2009 (293 cases); Period 2, 2010–2014 (353 cases); Period 3, 2015–2020 (566 cases). The trends for surgical technical complexity, oncological burden of the disease, use of the laparoscopic approach and use of techniques for hepatic hypertrophy were analyzed year by year. Uni- and multivariate analyses were performed in order to identify factors associated with inclusion to a laparoscopic approach and with long-term prognosis.

### 2.2. Preoperative Workup

Treatment strategies for each potential candidate to liver resection for CRLM were systematically evaluated at weekly multidisciplinary meetings, where liver and colorectal surgeons, radiologists, pathologists, hepatologists, specialists in nuclear medicine, medical oncologists and radiation oncologists assessed surgical resectability as well as the type of resection and technique.

Standard thoracoabdominal imaging (computed tomography and contrast-enhanced magnetic resonance) was routinely performed in all candidates prior to surgery, together with blood investigations, which included serum concentrations of tumor markers (carcinoembryonic antigen, Ca 19.9). Positron emission tomography (PET) was also performed in selected patients to rule out the presence of extrahepatic disease. Resectability of CRLM was defined by expert hepatobiliary surgeons as the ability to remove all liver disease while preserving an adequate volume of functional liver parenchyma, with adequate vascular inflow and outflow and maintaining the biliary drainage. Metastases were defined synchronous when they were present at the moment of primary tumor diagnosis. Preoperative chemotherapy was routinely administered in patients with initially unresectable liver metastases until conversion to resectability was achieved, and in patients with initially borderline/easily resectable liver disease with neoadjuvant intent unless a Clinical Risk Score (CRS) < 3 [22] (see later for details) was calculated. The same behavior was maintained over the whole study period.

### 2.3. Procedures

#### 2.3.1. Open Procedures

Xipho-supraumbilical incisions extending to the right subcostal area were performed. Major hepatectomies were defined as resection of three or more liver segments [23]. Intraoperative ultrasound was performed to assess lesion characteristics and the relationship to vascular structures. Whenever possible, primary vascular control was achieved prior to parenchymal transection. An ultrasonic dissector was used to fracture hepatocytes along the proposed line of transection, leaving arteries, veins, and bile ducts crossing the line of division intact; hepatic transection was completed with repeated, alternating use of an ultrasonic dissector and harmonic scalpel.

#### 2.3.2. Laparoscopic Procedures

For laparoscopic resections the patient was placed in the “French” position with the first surgeon standing between the patient’s legs and one assistant on each side. A four or five trocar configuration was used. The liver was evaluated by direct vision and intraoperative ultrasonography and the line of intended transection marked on its surface using diathermy. Hepatic transection was performed using the SonoSurg system (Olympus, Tokyo, Japan), which integrates an ultrasonic coagulating cutter and a conventional ultrasonic dissector. Vessels were sealed using bipolar forceps, clips or staplers, depending on their size. Resected specimens were placed in a retrieval bag and removed, without fragmentation, through enlargement of one of the port incisions or through a Pfannenstiel incision. Pringle’s maneuver was used as required to control intraoperative bleeding.

For combined resections for synchronous CRLM, all resections were performed by two separate teams, with the hepatobiliary unit performing liver surgery and the colorectal unit performing colorectal surgery [24].

### 2.4. Variables

Data on preoperative patient and disease characteristics, intraoperative findings and histopathological findings was collected. The Clinical Risk Score (CRS), as defined by the Memorial Sloan Kettering Cancer Group, was calculated for each patient. An index of difficulty for each operation was assessed using the Difficulty Score of Ban D et al. [25], which was developed in the setting of laparoscopic resections and takes into consideration five preoperative factors (tumor location, extent of liver resection, tumor size, proximity to major vessels and liver function). Intraoperative and postoperative outcomes, including morbidity and mortality, were evaluated. Postoperative complications were reviewed up to 90 days following liver resection and were graded retrospectively according to the Dindo-Clavien classification of surgical complications [26]. Mortality was defined as any death occurring during postoperative hospitalization or within 90 days after resection. Data regarding follow up, survival status and recurrence and type of recurrence were recorded. Three- and five-year overall survival (OS) and disease-free survival (DFS) were evaluated using the Kaplan−Meier method.

### 2.5. Statistical Methods

All variables were compared using the χ^2^ or Fisher’s exact test for categorical data, the Mann–Whitney U test for non-normally distributed continuous data, and Student’s *t*-test for normally distributed continuous variables. All data is expressed as a mean plus or minus the standard deviation or median and range. Uni- and multi-variate analyses were performed using the log rank test and the Cox proportional hazards. Cox regression was used to determine independent predictors of outcome, using postoperative morbidity as the dependent variable and significant factors (*p* < 0.05) on univariate analysis as covariates. Significance was defined as *p* < 0.05. All analyses were performed using the statistical package SPSS 18.0 (SPSS, Chicago, IL, USA).

## 3. Results

### 3.1. Participants and Recruitment Trends

**Figure 1:**
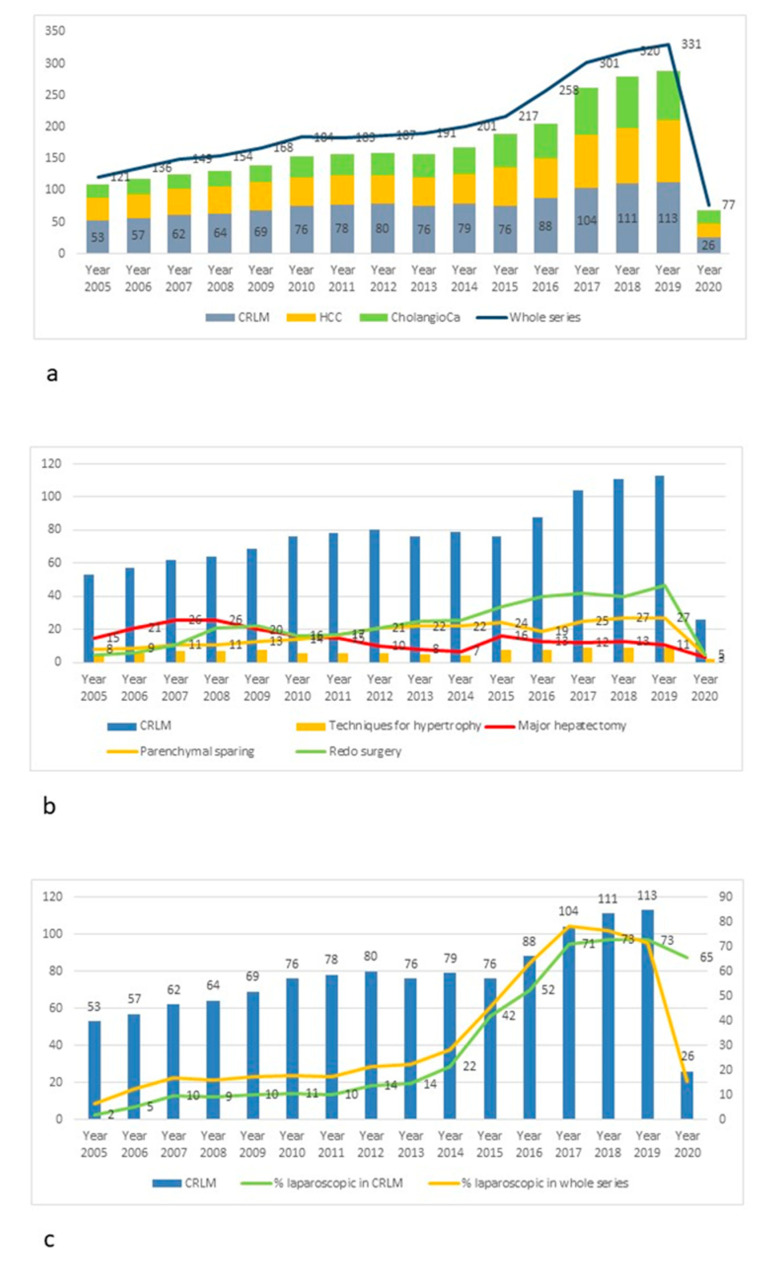
Time trends in colorectal liver metastases. (**a**) Breakdown of diagnosis for patients requiring liver resection stratified per year. (**b**) Surgery for colorectal liver metastases year by year, with trends of frequencies for major resections, parenchymal sparing resections, use of techniques for hypertrophy and redo surgery. (**c**) Trend in the use of laparoscopic approach within the whole series of liver resections and specifically in patients with colorectal liver metastases.

Between January 2005 and March 2020, 1212 liver resections for CRLM were performed, which constitute 36.5% of the whole series of resections performed in the same period. The number of liver resections for CRLM per year increased from 53 cases in 2005 to 113 cases in 2019 as shown in Figure 1a. A parallel increase was recorded both in the number of total liver resections per year and in the number of liver resections performed for primary liver neoplasms (hepatocellular carcinoma and cholangiocarcinoma). The proportion of resections for CRLM to the total number of liver resections ranged from 43.8% in 2005 to 34.1% in 2019, while the proportion of primary tumors to the whole series was 46.3% in 2005 and 53.2% in 2019. Table 1 reports the preoperative patient and disease characteristics in the whole series and after stratification according to period of recruitment. 80% of patients underwent neoadjuvant chemotherapy prior to surgery, with 44.3% receiving associated biological therapy. The use of biological therapies showed a significant increase over the study period (34.5% of patients in Period 1 and 51.8% in Period 3).

Primary tumor location was the right colon in 279 (23%) patients and the left colon in 351 (29%) patients, while 582 patients had a primary rectal cancer (48%). No significant differences were recorded between the three analyzed periods regarding the primary tumor location or T staging, grading and nodal status.

Disease presentation was synchronous and metachronous in 446 (36.8%) and 766 (63.2%) of cases respectively, with a significantly higher proportion of synchronous disease in Period 3 compared with Periods 2 and 1 (42% vs. 33.1% vs. 31.1% respectively, *p* = 0.04). An increasing number of cases with multifocal disease were detected in Period 3 compared with Periods 2 and 1 (65.1% vs. 51.8% vs. 44.7%, *p* = 0.02). Similarly, a higher proportion of redo hepatectomies (22.2%, 29.7% and 36.8% in Period 1, 2 and 3 respectively, *p* = 0.03) and patients with concurrent extrahepatic disease (4.8%, 10.8% and 14.3% in Period 1, 2 and 3 respectively, *p* = 0.03) were recruited in the most recent period. The median CRS of patients undergoing liver resection was 2 in Period 1, 3 in Period 2 and 4 in Period 3, which demonstrates a significant difference when specifically comparing Period 1 to Period 3 (*p* = 0.03). 

### 3.2. Surgery Characteristics and Outcomes (Technical Issues)

The number of patients requiring major hepatectomy was 232 (19.1%). Figure 1b shows the decreasing trend of major hepatectomies over recent years. There was indeed a significant difference in the incidence of major hepatectomies between Period 1, 2 and 3 (36.9% vs. 15.9% vs. 12%, *p* = 0.03). The use of techniques for induction of liver hypertrophy did not show any significant difference over the three periods (11.3% vs. 7.6% vs. 8% in Period 1, 2 and 3 respectively), as reported in Table 2. Conversely, a significant increase in the use of parenchymal sparing surgery was detected (17.7% in Period 1 versus 27.2% in Period 2 versus 22.4% in Period 3, *p* = 0.04). Median difficulty of procedures increased from 6 to 9 (*p* = 0.03) between Period 1 and Period 3.

33.8% of cases in the whole series were approached by laparoscopic technique, with an increasing trend recorded year by year (see Figure 1c), resulting in a significant difference for the use of minimally invasive technique among the three periods analyzed (7.8% in Period 1, 30.3% in Period 2 and 58.7% in Period 3, *p* = 0.02). Forty-seven out of 410 patients operated by laparoscopic approach required conversion to open approach, with no significant difference between periods in terms of conversion rate. The most frequent reasons for conversion were oncological concerns (22 cases, 46.8% of converted cases), bleeding (14 cases, 29.8% of converted cases) and adhesions (10 cases, 21.3% of converted cases).

Combined primary tumor resection for patients with synchronous CRLM were performed in 221 patients (18.2% of the whole series), with a similar incidence within the whole study period. An increasing proportion of patients with resectable extrahepatic disease were submitted to surgical treatment in Period 2 and 3 (4.8% and 6.4% respectively) compared to Period 1. Breakdown of the site of extrahepatic disease is shown in Table 2.

Table 3 reports the postoperative outcomes of patients for the whole series as well as stratified according to period of recruitment. No significant differences between Period 1, 2 and 3 were detected in terms of length of surgery, blood loss, use of Pringle maneuver and need for intraoperative blood transfusions.

The overall morbidity rate was 24.3% (16.6% minor complications (Dindo-Clavien I-II); 7.8% major complications (Dindo-Clavien III–V)), with no significant differences between groups. The 90-day mortality rate was 1.6% with 19 patients out of 1212 dying during the postoperative course. The most frequent causes of death were postoperative liver failure (8 patients), sepsis (6 patients) and cardiovascular accidents (3 patients). Median hospitalization time was five days (range 1–49) in the whole series.

Additionally, factors potentially affecting the choice of totally-laparoscopic approach were evaluated in the whole series of patients. On univariate analysis, 11 clinicopathological factors were analyzed (age; BMI; ASA Score; previous chemotherapy; CRS score; previous liver resection; combined primary tumor resection; number of liver lesions; extent of hepatectomy; difficulty score; period of inclusion). Of these factors, four were significantly associated with the use of the laparoscopic approach. When these significant factors were analyzed in multivariate analysis, only the number of liver lesions and the period of enrolment were found to be predictive factors for the laparoscopic approach. In particular, patients belonging to Period 3 had a 2.17 RR (95% Confidence Interval 1.95–3.07) to undergo the laparoscopic approach. The difficulty score did not show any significant correlation with the choice for a laparoscopic approach in the whole series.

### 3.3. Oncological Outcomes

**Figure 2:**
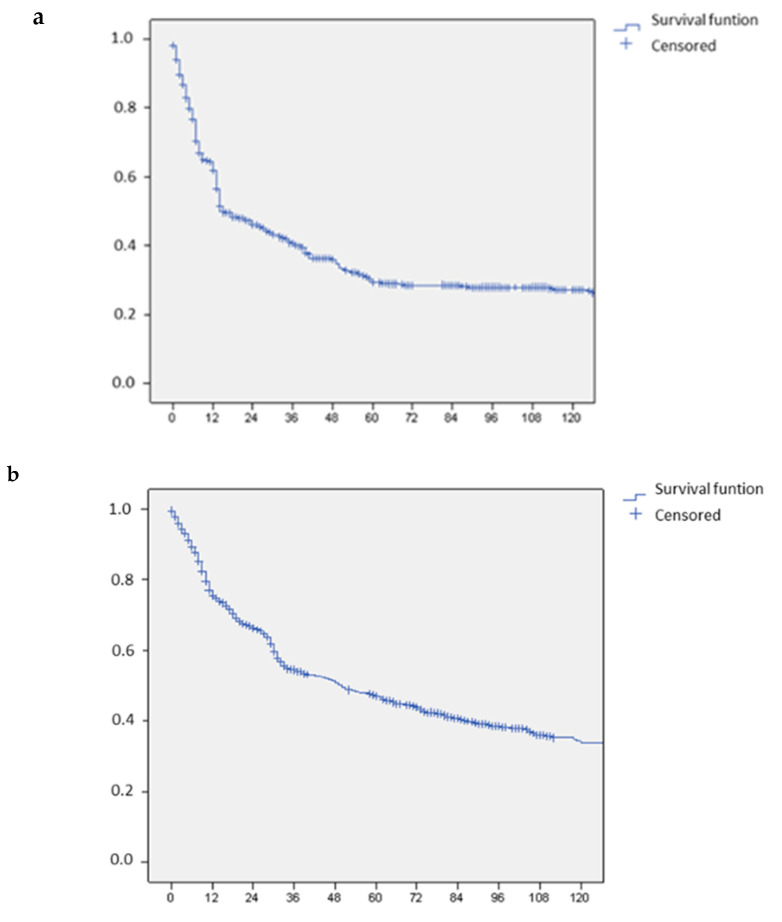
Long term outcome. (**a**) Recurrence free survival in the whole series of patients with colorectal liver metastases. (**b**) Overall survival in the whole series of patients with colorectal liver metastases.

**Figure 3:**
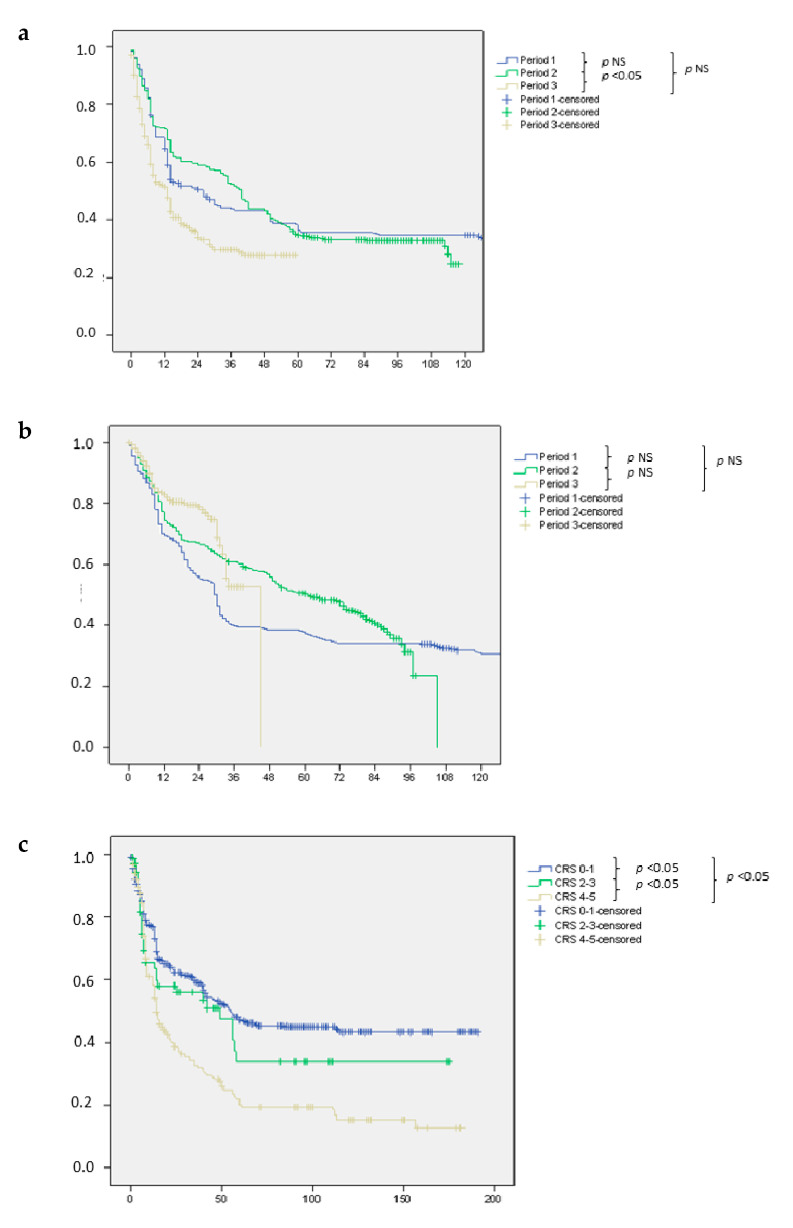

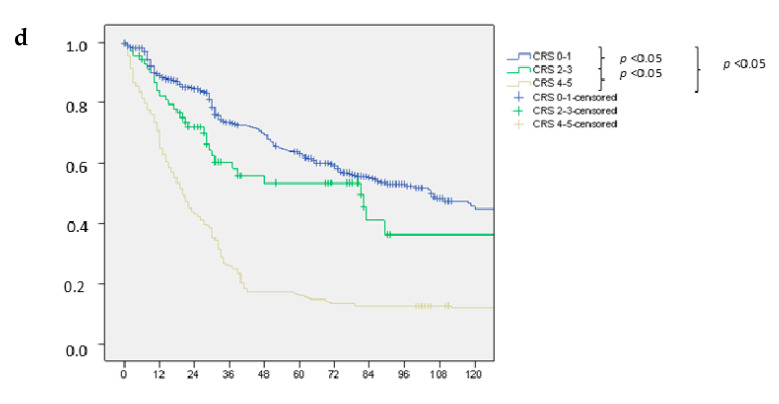
Long term outcome according to period and Clinical Risk Score (CRS). (**a**) Recurrence free survival in the series of patients with colorectal liver metastases stratified according to period of recruitment. (**b**) Overall survival in the series of patients with colorectal liver metastases stratified according to period of recruitment. (**c**) Recurrence free survival in the series of patients with colorectal liver metastases stratified according to CRS. (**d**) Overall survival in the series of patients with colorectal liver metastases stratified according to CRS.

The rate of R0 resections in the whole series was 92.1%, with a lower incidence of R1 margins in Period 1 compared to Period 2 and 3. Depth of resection margin was on median 5 mm in the whole series, with a significant reduction recorded in Period 3 (median: 4 mm) compared to Period 2 (median: 8 mm) and Period 1 (median: 9 mm), *p* = 0.03. The median overall survival was 58 months (range: 6–115). Median disease free survival was 36 months (range: 6–115), while disease recurrence occurred in 50.5% of patients. Table 4 reports the pattern of recurrence and type of treatment for patients who experienced intrahepatic recurrence. Kaplan Meier curves of overall and disease free survival are shown in Figure 2a,b. Overall and disease free survival in each of the three periods analyzed are reported and after stratification according to the CRS score, in Figure 3a,3b and 3c,3d respectively.

Factors potentially affecting disease free survival were evaluated in the entire series of patients. On univariate analysis, 20 clinicopathological factors were analyzed (age; sex; BMI; ASA Score; perioperative chemotherapy; CRS score; primary tumor location; primary tumor staging; primary tumor nodal involvement; KRAS status; synchronous presentation; redo surgery; extrahepatic disease; number of liver lesions; dimension of liver lesions; extent of hepatectomy; resection margin; intraoperative blood loss; postoperative complications; approach to liver resection). Six of these factors were shown to be significantly associated with operative outcome. Multivariate analysis revealed that the CRS score, primary tumor location, perioperative chemotherapy and presence of complications were independent prognostic factors for disease free survival.

## 4. Discussion

Literature regarding CRLM has specifically focused on technical and onco-surgical aspects in recent years. Among technical advances, over the recent years there has been the establishment and diffusion of laparoscopic liver surgery [7,8], the value of R1 resection has emerged [9,10] and the issue of residual liver volume has been addressed through the implementation of two-stage hepatectomies [11,12] and ultrasound-guided parenchymal sparing resections [13]. Among onco-surgical aspects, on the other hand, the impact of Chemotherapy Associated Liver Injury (CALI) [14,15] and disappearing metastases has been reported [16], the appropriate management of extrahepatic disease has been clarified [17] and the correct balance between timing of surgery and oncological treatment has been established [18]. Finally, the analysis of prognostic factors for the selection and stratification of candidates has been identified as a topic of paramount importance [13,19].

The absolute number of liver resections for CRLMs has progressively grown over recent years, following the growing availability of technological and structural resources [7,27]. However, the same trend of growth was recorded for all malignant tumours of the liver [28,29,30,31], resulting in an overall increase in the annual volume of liver resections and in a similar proportion of surgical indications among different periods.

When focusing on disease with characteristics of high technical complexity [25] and high CRS [22], a different trend was observed. Aside from the increase in the absolute number of cases, which parallels the policy of broader inclusion to surgical treatment, the proportion of cases with high compared to low complexity and cases with high CRS compared to those with lower CRS significantly increased. Indeed, thanks to the wider and large-scale availability of advanced technologies and following the accessibility to training courses in open and minimally invasive hepatic surgery [32], cases with a low profile of complexity are currently managed with an adequate safety profile in centers with a general surgery unit and low volumes of hepatic surgery. On the contrary, cases with borderline oncological indications or with technical challenges are referred and managed within tertiary referral centers. The hub and spoke model in liver surgery [33] was therefore stably implemented, which ensures active and mutual collaboration between centers and surgeons and promotes a collegial discussion of each case for its subsequent allocation to the hub or spoke center according to criteria of complexity and technical feasibility.

The increase in complex cases corresponds to a significant increase in the use of techniques for induction of hepatic hypertrophy (PVE, Two stage Hepatectomy, ALPPS), which demonstrated greatest benefit in the setting of CRLM, as reported in several series specifically designed to address this topic [11,12,34]. Similarly, the use of parenchymal sparing resections with mandatory and continual use of intraoperative ultrasound [10,35] has increased the resectability rate for patients with multiple and bilobar lesions in recent years. Interestingly, multiple parenchymal sparing resections have increased in parallel with the use of intraoperative ablation methods. For lesions < 2 cm in size, ablation seems to constitute an oncologically effective alternative to resection in selected cases [36], although the overlap in terms of long-term outcome is the subject of an ongoing RCT [37]. Anyway, results from the CLOCC study [38]—reporting the outcome of 119 patients with unresectable colorectal liver metastases (*n* < 10 and no extrahepatic disease) receiving systemic treatment alone or systemic treatment plus aggressive local treatment by radiofrequency ablation ± resection—demonstrated that aggressive local treatment can prolong OS in patients with unresectable colorectal liver metastases, therefore providing the cultural background to support the use of ablation.

No differences were seen in terms of local recurrence for R1 patients over the three period: it is likely that—despite the indication to perform R1 resection was expanded over years due to the increasing number of patients with borderline resectable disease—the scenario to consider R1 was the same. In author’s experience indeed, R1 can be an option only in patients who are otherwise unresectable (for instance for lesions close to major branches of the liver remnant). The upward trend in parenchymal sparing resections did not correspond to a proportional increase in R1 resections, which might be considered for otherwise irresectable patients, but do not replace the value of R0 resections, which are still considered the gold standard of treatment. Furthermore, an inverse relationship between the number of parenchymal sparing resections and major hepatectomies (which maintain a stable absolute number but represent a lower proportion of all resections) was recorded, with the rationale of allowing the possibility of redo surgery in cases of resectable intrahepatic recurrences.

In the most recent period, a significantly higher incidence of synchronous metastases (taking into consideration both lesions managed within a combined surgery with the primary tumor as well as lesions managed with the liver-first or colon-first strategy) [39] compared to the initial period analyzed was recorded. Reasons for this can be both onco-surgical and strategic. Regarding onco-surgical issues, it is likely that modern chemotherapy regimens and advanced surgical strategies have allowed an increasing number of patients with unresectable (or borderline resectable) disease at presentation to be converted to resectability [40]; consequently, the percentage of patients with synchronous CRLM managed at dedicated centres has recently increased. Regarding strategic issues, it is likely that metachronous disease, which by definition is diagnosed during follow-up and which is therefore generally detected when a lower burden of intrahepatic disease is present, could be adequately managed even in centers with low-volume of hepatic surgery.

Despite the recruitment of a population of patients with an increasing complexity in terms of oncological burden, the long-term outcome was globally comparable in the three time periods analyzed, while a worse DFS was recorded in the last period compared with the first. However, after stratification of patients by CRS score, these differences were no longer evident and the DFS became comparable among the three periods.

Within patients with the same CRS, when comparing period 1 to period 3, patients operated in the most recent period had a significant advantage in terms of DFS. Although it is beyond the scope of this study to investigate the reasons for this improvement, a beneficial role of newer biological drugs whose targeted use has enhanced the results of modern chemotherapy schemes may be speculated [41,42]. In fact, although patients in period 1 underwent a similar number of CT cycles compared to patients in period 3, patients in period 1 were less frequently treated with biological drugs and paradoxically underwent a greater number of cycles of neoadjuvant CT. It is likely that the detrimental effect of CALI on post-operative outcomes was not fully appreciated, even in the setting of the multidisciplinary team, in the initial period and possibly, patients were even referred later for surgical evaluation in the past compared with today. At the same time, better control of postoperative morbidity can be seen in the most recent series. This has a favourable impact on the risk of neoplastic recurrence and even allows for earlier return to an adjuvant chemotherapy program [7,18]. The experience gained over the years and the currently available evidence guiding clinical behavior in the context of CRLMs should have smoothed out the differences in terms of treatment schedules worldwide, even when complex disease (bilobar metastases, recurrent lesions) is analysed. On the other hand, it is possible that some differences can still be detected between high and low volume centers within the same country: this is the rationale to support the need to manage complex disease in referral settings.

The reduction in postoperative morbidity seen in recent times can be traced back to two main factors. On one hand, a more mature indication for and management of hepatic hypertrophy techniques, which, at present, combine volumetric and functional assessment of the residual hepatic parenchyma [43]. This evaluation allows the patient to be managed using an increasingly holistic and safe preoperative optimization strategy. The current protocol for the management of candidates to two-stage hepatectomy for multiple and bilobar lesions follows the conventional two stage hepatectomy as described by Adam [44] and then modified by Jaeck [45] as the standard indication, while ALPPS is considered only for a selected group of patients with high risk of interstage progression and with a very small FLR. On the other hand, the introduction of the minimally invasive approach has certainly contributed to improved outcomes, as evidenced by the reduced blood loss, postoperative morbidity reduction and shortening of postoperative stay [7]. This finding is likely linked to a reduction in biological impairment and surgical stress [7,46].

Laparoscopy has spread considerably in recent years in all areas of oncological liver surgery [7,27,28,29,30,31,32]. The inversion of the proportion of cases between the open and laparoscopic approach has also occurred in the context of CRLM, even if with a slower speed compared with hepatocellular cancer [28]. Indeed, in order to overcome the technical challenges of laparoscopy in a stepwise fashion, cases with a lower profile of complexity were enrolled at the beginning of the experience [47]. This “clashed” with the inverse trend of the overall series, since in the later periods more cases of CRLM with increasing complexity and higher neoplastic burden were enrolled to expand the chance of cure (especially in this referral center setting). After having overcome some technical challenges, the laparoscopic approach has gained momentum in the field of CRLM [7]. In the multivariate analysis indeed, the late period of inclusion in the series, the first liver resection (no redo surgery) and a number of lesions < 3 were predictive factors for the laparoscopic approach.

The presence of a multidisciplinary management group for the oncosurgical pathway of patients is the strategic key for CRLM: there are some interesting reports in the literature that demonstrate how surgical resectability—especially when borderline resectable disease is present—is largely underestimated when evaluated solely from the oncological viewpoint, leading to failed opportunities to provide potential cure to a subgroup of patients [20,21,48].

This series reports how the treatment of CRLMs has undergone radical changes in recent years and that the multidisciplinary group, once established and consolidated, must make a continuous effort to update and discuss indications and approaches, in line with evidence from the literature that remains in continuous evolution. This analysis confirms on a large scale how molecular, translational and clinical studies that have been developed to analyze a specific aspect of this complex disease have an actual clinical counterpart and have deeply modified the practice of surgeons.

## 5. Conclusions

The cultural background, the maturation of technical expertise and the consolidation of the multidisciplinary team have resulted in safe expansion of the possibility to offer a curative opportunity to patients, while continuously implementing into clinical practice evidence provided by the literature.

## Figures and Tables

**Table 1:** Preoperative characteristics of patients and disease in the whole series and stratified according to period of recruitment.

Variables		Whole Series	Period 1 (2004–2009)	Period 2 (2010–2015)	Period 3 (2015–2020)	a vs. b	a vs. c	b vs. c	a vs. b vs. c
		1212	293	353	566				
Age, median (range)	years	60 (37–80)	59 (32–80)	62 (31–83)	61 (26–89)	NS	NS	NS	NS
Gender, *n* (%)						NS	NS	NS	NS
	Male	678 (55.9)	166 (56.7)	191 (54.1)	321 (56.6)				
	Female	534 (44.1)	127 (43.3)	162 (45.9)	245 (43.4)				
ASA Score, *n* (%)						NS	0.05	0.04	0.04
	I/II	773 (63.8)	197 (67.2)	241 (68.3)	335 (59.2)				
	III/IV	439 (36.2)	96 (32.8)	112 (31.7)	231 (40.8)				
Neoadjuvant CT, *n* (%)		970 (80)	211 (72)	299 (84.7)	460 (81.2)	0.03	0.04	NS	0.03
CT regimen, *n* (%)						0.04	NS	NS	0.04
	Oxaliplatin based	583 (48.1)	123 (42)	171 (48.4)	289 (51.1)				
	Irinotecan based	439 (36.2)	76 (25.9)	155 (43.9)	208 (36.7)				
	Associated biological therapy	537 (44.3)	89 (30.4)	155 (43.9)	293 (51.8)	0.02	0.01	0.05	0.03
Number of CT cycles, median (range)		6 (1–23)	9 (1–16)	7 (1–23)	6 (3–19)	0.04	0.03	NS	0.05
Associated comorbidites, *n* (%)		582 (48)	101 (34.5)	167 (47.3)	314 (55.5)	0.03	0.04	NS	0.03
Features of non-tumorous parenchyma, *n* (%)						NS	0.03	NS	0.04
	Normal	538 (44.4)	95 (32.4)	167 (47.3)	276 (48.8)				
	Steatosis	252 (20.8)	77 (26.3)	50 (14.2)	125 (22.1)				
	CALI	422 (34.8)	121 (41.3)	136 (38.5)	165 (29.1)				
Primary tumor location, *n* (%)						NS	NS	NS	NS
	Right colon	279 (23)	81 (27.6)	96 (27.2)	102 (18)				
	Left colon	351 (29)	94 (32.1)	108 (30.6)	149 (26.4)				
	Rectum	582 (48)	118 (40.3)	149 (42.2)	315 (55.6)				
Staging, *n* (%)						NS	NS	NS	NS
	T1	47 (3.9)	11 (3.8)	18 (5.1)	18 (3.2)				
	T2	534 (44.1)	127 (43.3)	154 (43.6)	253 (44.8)				
	T3	530 (43.7)	131 (44.7)	145 (41.1)	254 (44.8)				
	T4	101 (8.3)	24 (8.2)	36 (10.2)	41 (7.2)				
Grading, *n* (%)						NS	NS	NS	NS
	G1	96 (7.9)	27 (9.2)	31 (8.8)	38 (6.7)				
	G2	922 (76.1)	201 (68.6)	266 (75.4)	455 (80.4)				
	G3	194 (16)	65 (22.2)	56 (15.9)	73 (12.9)				
Nodal status, *n* (%)						NS	NS	NS	NS
	N0	583 (48.1)	141 (48.1)	172 (48.7)	270 (47.7)				
	N1	488 (40.3)	117 (39.9)	134 (38)	237 (41.9)				
	N2	141 (11.6)	35 (11.9)	47 (13.3)	59 (10.4)				
Presentation, *n* (%)						NS	0.04	0.04	0.04
	Synchronous	446 (36.8)	91 (31.1)	117 (33.1)	238 (42)				
	Metachronous	766 (63.2)	202 (68.9)	236 (66.9)	328 (58)				
Number of liver lesions, median (range)		3 (1–44)	2 (1–12)	2(1–44)	3 (1–32)	NS	NS	NS	NS
Nodularity, *n* (%)						NS	0.02	0.01	0.02
	Monofocal	530 (43.7)	162 (55.3)	170 (48.2)	198 (34.9)				
	Multifocal	682 (56.3)	131 (44.7)	183 (51.8)	368 (65.1)				
Lobe distibution of metastases, *n* (%)						NS	0.03	0.05	0.04
	Unilobar	630 (52)	184 (62.8)	191 (54.1)	255 (45.1)				
	Bilobar	582 (48)	109 (37.2)	162 (45.9)	311 (54.9)				
Redo liver surgery, *n* (%)		378 (31.2)	65 (22.2)	105 (29.7)	208 (36.8)	0.04	0.01	0.04	0.03
Extrahepatic disease, *n* (%)		133 (11)	14 (4.8)	30 (10.8)	81 (14.3)	0.04	0.01	0.03	0.03
Liver met diameter, median (range)		2.9 (0.5–11)	2.7 (0.5–9)	2.9 (0.5–11)	2.8 (0.5–13)	NS	NS	NS	NS
Clinical Risk Score, median (range)		3 (1–5)	2 (1–5)	3 (1–5)	4 (1–5)	NS	0.03	NS	0.05
CEA level, median (range)		35.6 (2–299)	31.9 (2–135)	35.6 (2–276)	44.5 (2–1045)	NS	NS	NS	NS

**Table 2:** Intraoperative characteristics of procedures in the whole series and stratified according to period of recruitment.

Variables		Whole Series	Period 1 (2004–2009)	Period 2 (2010–2015)	Period 3 (2015–2020)	a vs. b	a vs. c	b vs. c	a vs. b vs. c
		1212	293	353	566				
Extent of liver resection, *n* (%)						0.01	0.01	NS	0.03
	Major	232 (19.1)	108 (36.9)	56 (15.9)	68 (12)				
	Minor	980 (80.9)	185 (63.1)	297 (84.1)	498 (88)				
Technique for liver hypertrophy, *n* (%)		105 (8.7)	33 (11.3)	27 (7.6)	45 (8)	NS	NS	NS	NS
	PVE alone	11 (0.9)	2 (0.7)	4 (1.1)	5 (0.9)				
	Two stage	76 (6.3)	31 (10.6)	20 (5.7)	25 (4.4)				
	ALPPS	22 (1.8)	0	7 (2.0)	15 (2.7)				
Parenchymal sparing surgery, *n* (%)		275 (22.7)	52 (17.7)	96 (27.2)	127 (22.4)	0.03	0.04	NS	0.04
Median difficulty		8 (3–10)	6 (3–10)	7 (3–10)	9 (3–10)	NS	0.03	0.05	0.03
Laparoscopic approach, *n* (%)		410 (33.8)	23 (7.8)	107 (30.3)	332 (58.7)	0.03	0.01	0.03	0.02
Conversion, *n* (%)		47 (11.5)	3 (13)	13 (12.1)	32 (9.6)	NS	NS	NS	NS
Primary tumor resection, *n* (%)		221 (18.2)	56 (19.1)	72 (20.4)	93 (16.4)	NS	NS	NS	NS
Intraoperative ablation, *n* (%)		145 (12)	21 (7.2)	50 (14.2)	74 (13.1)	0.04	0.05	NS	0.04
Margin, *n* (%)						0.03	0.05	NS	0.04
	R0	1116 (92.1)	280 (95.6)	316 (89.5)	520 (91.9)				
	R1	96 (7.9)	13 (4.4)	37 (10.5)	46 (8.1)				
Extrahepatic disease removal, *n* (%)		62 (5.1)	9 (3.1)	17 (4.8)	36 (6.4)	NS	0.04	0.05	0.05
	Peritoneal	21 (1.7)	3 (1.0)	7 (2)	11 (1.9)				
	Nodal	52 (4.3)	7 (2.4)	21 (5.9)	24 (4.2)				
	Pulmonary	7 (0.6)	1 (0.3)	3 (0.8)	4 (0.7)				
									

**Table 3:** Intra and postoperative outcome of procedures in the whole series and stratified according to period of recruitment.

Variables		Whole Series	Period 1 (2004–2009)	Period 2 (2010–2015)	Period 3 (2015–2020)	a vs. b	a vs. c	b vs. c	a vs. b vs. c
		1212	293	353	566				
Operating time, median (range)	Minutes	240 (150–640)	300 (220–640)	220 (180–510)	250 (150–590)	NS	NS	NS	NS
Blood loss, median (range)	mL	280 (100–1600)	400 (100–1200)	250 (100–1400)	300 (100–1600)	0.04	NS	NS	NS
Pringle maneuvre, *n* (%)		1053 (86.9)	261 (89.1)	309 (87.5)	483 (85.3)	NS	NS	NS	NS
Length of Pringle manouevre, median (range)	Minutes	40 (15–135)	30 (10–110)	55 (10–120)	45 (10–135)	0.04	0.05	NS	0.05
Intraoperative blood transfusion, *n* (%)		96 (7.9)	21 (7.2)	34 (9.6)	41 (7.2)	NS	NS	NS	NS
Depth of liver margin, median (range)	mm	5 (0–11)	9 (0–22)	8 (0–19)	4 (0–11)	NS	0.02	0.04	0.03
Time to first flatus, median (range)	days	3 (2–6)	3 (2–6)	3 (2–6)	3 (2–6)	NS	NS	NS	NS
Return to diet, median (range)	days	1 (0–6)	1 (0–5)	1 (0–6)	1 (0–4)	NS	NS	NS	NS
Morbidity, *n* (%)		295 (24.3)	75 (25.6)	90 (25.4)	130 (22.9)	NS	NS	NS	NS
Minor Morbidity (Dindo-Clavien I–II)		201 (16.6)	55 (18.7)	56 (15.9)	90 (15.9)	NS	NS	NS	NS
Major Morbidity (Dindo-Clavien III–V)		94 (7.8)	20 (6.8)	34 (9.6)	40 (7.1)	NS	NS	NS	NS
Mortality, *n* (%)		19 (1.6)	5 (1.7)	7 (1.9)	7 (1.2)	NS	NS	NS	NS
Postoperative transfusions, *n* (%)		178 (14.7)	51 (17.4)	63 (17.8)	64 (11.3)	NS	0.04	0.05	0.05
Total transfusions, *n* (%)		223 (18.4)	61 (20.8)	72 (20.4)	90 (15.9)	NS	NS	NS	NS
Lenght of postoperative stay, median (range)	days	5 (1–49)	6 (1–38)	5 (1–49)	5 (1–43)	NS	NS	NS	NS

**Table 4:** Long-term outcome according to treatment group.

Variables		Whole Series	Period 1 (2004–2009)	Period 2 (2010–2015)	Period 3 (2015–2020)	a vs. b	a vs. c	b vs. c	a vs. b vs. c
		1212	293	353	566				
Death, *n* (%)		315 (26)	104 (35.5)	108 (30.6)	103 (18.2)	0.01	0.01	0.01	0.01
Cause of death, *n* (%)						NS	NS	NS	NS
	Tumor progression	308 (97.8)	101 (97.1)	106 (98.1)	101 (98.1)				
	Other	7 (2.2)	3 (2.9)	2 (1.9)	2 (1.9)				
Disease recurrence, *n* (%)		612 (50.5)	189 (64.5)	207 (58.6)	216 (38.2)	0.01	0.01	0.01	0.01
Modality of recurrence, *n* (%) *						NS	NS	NS	NS
	Intrahepatic	255 (41.7)	86 (45.5)	102 (49.3)	67 (31)				
	Extrahepatic	156 (25.5)	43 (22.8)	36 (17.4)	77 (35.6)				
	Extrahepatic + intrahepatic	201 (32.8)	60 (31.7)	69 (33.3)	72 (33.3)				
Therapy of intrahepatic recurrence, *n* (%) **						NS	NS	NS	NS
	Re-resection	189 (41.4)	71 (48.6)	69 (40.4)	49 (35.3)				
	Local treatments	49 (10.7)	17 (11.6)	18 (10.5)	14 (10.1)				
	Medical therapy	237 (52)	89 (61)	76 (44.4)	72 (51.8)				

* Percentage is calculated by dividing the number of patients with recurrence by total number of recurrences; ** Percentage is calculated by dividing the number of patients with recurrence by total number of intrahepatic recurrences.

## Data Availability

The data presented in this study are available on request from the corresponding author. The data are not publicly available due to privacy reasons.
